# Extraction of Lightweight *Platanus orientalis* L. Fruit’s Stem Fiber and Determination of Its Mechanical and Physico-Chemical Properties and Potential of Its Use in Composites

**DOI:** 10.3390/polym16050657

**Published:** 2024-02-28

**Authors:** Ali Ihsan Kaya

**Affiliations:** Department of Mechanical Engineering, Engineering Faculty, Adıyaman University, 02040 Adıyaman, Turkey; alikaya@adiyaman.edu.tr

**Keywords:** *Platanus orientalis* L., mechanical properties, extracted new fiber, composite fibers, sustainability, morphology and chemistry of *Platanus orientalis* L.

## Abstract

Natural fibers extracted from plants are preferred as an alternative to synthetic products. The main reasons for this preference are their affordable cost, light weight and good mechanical properties. However, finding new natural raw materials is challenging due to growth limitations in different geographical areas. *Platanus orientalis* L. (Eastern plane tree) is a tree with abundant fruits that can grow in many regions of the world. The aim of this study was to determine the mechanical (tensile strength, tensile modulus, elongation), physical (density, fiber diameter) and chemical (cellulose, hemicellulose and lignin) properties of *Platanus orientalis* L. fruit’s stem by fiber extraction from the stems of the tree. It was determined that the extracted fiber had good mechanical properties and cellulose content of 42.03%. As a result of thermogravimetric analysis, it was determined that the plane tree fruit’s stem fiber had thermal resistance of up to 299 °C. The tensile strength value was 157.76 MPa, the tensile modulus value was 1.39 GPa and the elongation value was 22.01%. It was determined that it is suitable for use in fiber reinforcement in thermoplastic-based composites at temperatures below 299 °C. According to the results obtained by the mechanical, chemical and physical analysis of *Platanus orientalis* L. fruit’s stem fiber (PoLfs), it could be recommended as a suitable alternative as a reinforcing fiber in thermoplastic and thermoset composites.

## 1. Introduction

Belonging to the Platanaceae family, the plane tree (*Platanus orinetalis* L.) takes on a rounded form as it ages and a pyramidal form when it is younger. The plane tree is a large deciduous tree with a very thick and short trunk that spreads thick and long branches upward and to the sides. It grows to a height of about 30–50 m and a diameter of 1–2.5 m [1,2]. Older trunks have deeply fractured bark that is covered in tiny scales that do not fall off over time. The lobes on leaves with three to seven lobes are coarsely toothed or entire-edged, and the grooves between them are narrowly angled and penetrate all the way to the middle of the leaf blade, even near the petiole. The leaves are deeply dissected. The lengths of the petioles are 2.5–7.5 cm [1]. The plane tree has ball-shaped fruits. Fruits stem from these “globose heads”; the female flowers of the plant develop into walnut-sized beans following pollination [3]. Three to seven fruits hang together on a long stem (13 to 19 cm). The tree originated in Western Asia and Southeast Europe. Its distribution area is very wide, ranging from sea level to an altitude up to 1100 m in the majority of Turkey. It is primarily found near streams, river banks, river deltas, seepage and gravelly slopes [4]. Due to its long lifespan, the plane tree is particularly favored [5]. While some of these species’ very old members have been lost to history, others have been preserved as natural monuments [4]. It is reported in the literature that the Oriental plane tree is used in folk medicine [5]. For example, Oriental plane roots are reported to be used as an antidote for snake bites, while the leaves are used to treat a variety of inflammatory, rheumatic, gastrointestinal and dermatological disorders. However, it has also been reported that sycamore pollen is a source of airborne allergens and can cause reactions such as asthma, allergic rhinitis and allergic conjunctivitis [6].

The study by Janković et al., 2018 [7] aimed to examine a potential solid sorbent for a sorption process for harmful gases like CO_2_; the purpose of the study was to choose experimental techniques for the characterization analysis of raw and pyrolyzed samples of plane tree (*Platanus orientalis* L.) seeds and to assess the results. A study by Dodevski et al., 2020 was reported to characterize and analyze the activated carbons produced by the horizontal tubular reactor-based pyrolysis of plane tree seeds [8]. As they stated, since woody-based biomass and wood charcoals can also be used for cadmium adsorption, their research is significant. In particular, it is possible to use the ash and leaves of *Platanus orientalis* L. (PoLfs) as adsorbents to remove cadmium from aqueous solutions. The purpose of another study [9] was to determine how well raw Platanus fruit fibers adsorb various kinds and concentrations of oil in water. As a result of the investigation, it was determined that the temperature of the oil, the amount, the concentration and the surface characteristics of the fibers all affected how well they removed oil from water. Güler et al., 2017 [5], in their study, aimed to ascertain the profile of volatile compounds present in *Platanus orientalis* leaves and stated that *Platanus orientalis* leaves contained 140 different volatile compounds in total. Yang et al., 2016 [10] successfully used Platanus fruit fibers, which have distinctive hollow tubular structures, for the first time to create effective oil sorbents by chemically modifying them with acetic anhydride. Additionally, the researchers not only reported on the utilization of plane tree fibers to create porous pipe walls for tubular structures but also proposed an inexpensive and effective oil-absorbing substance created by a hydrophobic alteration process [11]. By utilizing the precursor of sycamore fruit seeds and altering the carbonization conditions, a novel type of biomass hard carbon was produced [12], and the authors stated that sycamore fruit seed hard carbon anode material has practical uses in the industrialization of sodium-ion batteries, in addition to having the potential to replace lithium-ion batteries due to its easy preparation, cheap cost and the plentiful supply of raw materials. Some other studies reported on the creation of a dye removal adsorbent and solar evaporator by utilizing 3D spherical carbonized fibers and fruits [13,14]. By using short natural fibers from plane tree (*Platanus orientalis* L.) fruits, Atabek Savas 2022 [2] examined the mechanical characteristics of polypropylene composites. Moreover, it is possible to utilize numerical methods to evaluate the mechanical performance of the new extracted fibers in the literature [15] instead of time-consuming and expensive experimental techniques. Considering earlier studies, it is evident that most of the studies in the literature have focused on the fruit of the plane tree or its fiber. However, in this study, unlike the literature, the characterization of the fibers of the fruit stem of the plane tree has been investigated instead of the fruit of the plane tree.

In order to promote sustainability and a greener environment, there has been a lot of interest worldwide in the use of natural fibers as substitute materials in various industrial sectors. In particular, the problems caused by global warming are pushing the world’s industries to search for more sustainable solutions. For this reason, there are considerable scientific studies on possible composites created by combining natural fibers or polymers. In particular, cellulosic-based characterization studies are of great importance for this reason because it is even argued that this century will be the century of cellulose [16]. Composite materials are new structures formed by combining two materials in the macro dimension. The main purpose here is to provide maximum benefit by bringing the properties of two materials together [17,18]. Polymeric resins have been combined with various natural fibers, such as kenaf, sugar palm, flax, jute, hemp, etc., to create new materials known as natural fiber composites [19,20,21,22,23]. According to this perspective, the use of natural fiber composites has attracted the interest of material scientists and engineers because of the benefits they provide [24,25,26,27,28,29,30,31].

Numerous fields focused on mechanical, medical, and industrial applications are affected by the wide range of applications of cellulose [32]. Cellulose is utilized in the manufacturing of degradable polymers by reinforcing matrixes. Additionally, applications for cellulose include packaging, tissue engineering, electronics, optics, sensors, pharmaceuticals, cosmetics, insulation, water filtration, hygienic uses and vascular grafts [33,34,35]. Moreover, chemical treatment processes (alkalization, enzymes, etc.) can be applied to the surface of natural fibers to increase fiber/matrix surface compatibility. It is thought that fiber/matrix surface compatibility will improve by removing the impurities on the surface as a result of increasing the existing cellulose ratio of natural fiber with chemical treatment processes [36]. Therefore, cellulosic fiber characterization studies are in great demand as they have the potential to be used in different industries, and the number of studies on characterization continue to increase in the literature [37,38,39].

Every year, a single Platanus tree produces 300 spherical fruits and, accordingly, stem fibers on average. These fruits’ stems are typically disposed of as waste in the environment and as potential sources of allergies for vulnerable groups [2,40]. Vast amounts of plane tree fruits have been scrapped as bio-waste material, despite the fact that plane trees can be grown anywhere in the world and used for a variety of purposes (furniture, flooring, medicine, printing, the greening of streets and gardens, etc.). They produce harmful gas emissions and carbon dioxide emissions when disposed of in landfills, and they are inedible to humans [11,41]. However, both plane tree fruit and fruit stalks, which become waste as a result of seasonal transitions, have great potential as fiber materials. Therefore, in this study, the fiber characterization of the plane tree’s fruit stem, which has been ignored so far, was investigated instead of the plane tree fruit, which is frequently studied in the literature. Density determination and physical characterization determination of fruit stem fibers obtained after stems were kept in water for about 3 weeks with the natural decay of the bark component were carried out. In addition, thermogravimetric analysis (TGA), X-ray diffraction (XRD), scanning electron microscopy (SEM) and Fourier transform infrared spectroscopy (FTIR) tests were applied for chemical characterization.

## 2. Materials and Methods

### 2.1. Fiber Extraction

**Figure 1:**
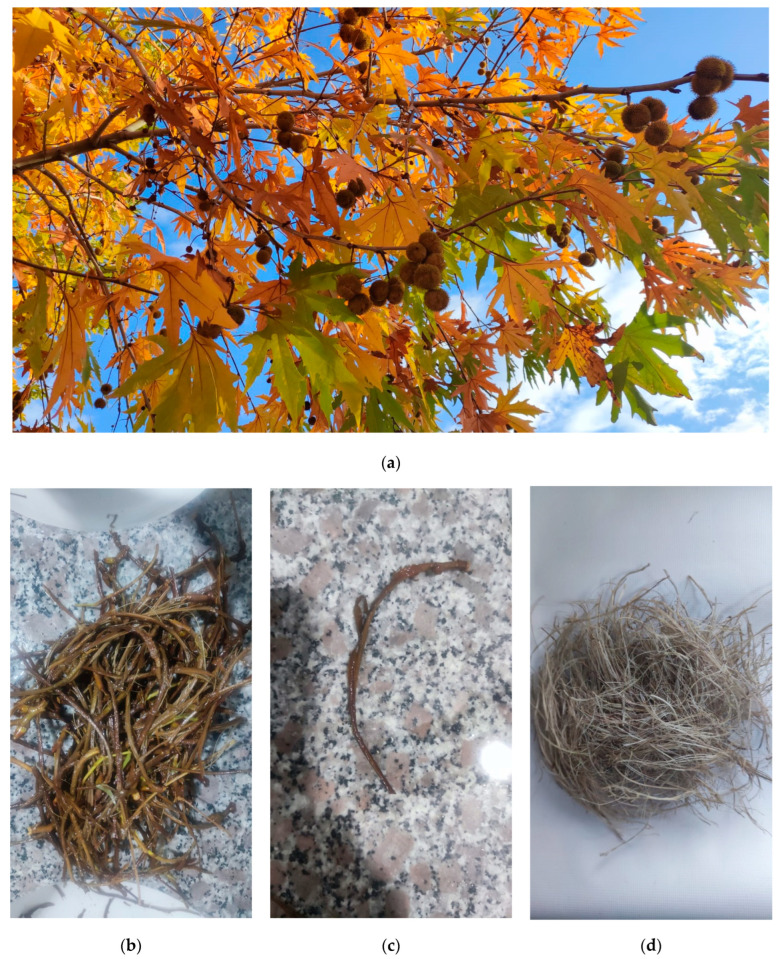
*Platanus orientalis* L. fruits and its stems (**a**), after the decaying process of stems (**b**), single stem of the plane tree fruit with rotted bark in water to be extracted for fibers (**c**), extracted fibers after washing (**d**).

*Platanus orientalis* L. fruit stems, from which fibers were to be extracted, were collected from the plane trees in early November when the fruits were at their ripest. The ripest state of the fruits and hence the stems could be ascertained from the brown color of the fruits in Figure 1a and the yellowing of the leaves. The fruits were harvested from the plane trees (Figure 1a) located within the borders of Altınşehir Campus of Adıyaman University. A latitude of 37.744331 and a longitude of 38.229885 are the location specifications for the campus. The altitude (height above sea level) of the campus is 670 m, and the GPS coordinates are 37°44′39.5916″ and 38°13′47.5860″. After separating the harvested fruits and stems with scissors, the fruit stems were left to decompose in a bucket filled with water for three weeks. The time of decay was determined by observing the decay of the stem bark (Figure 1b). The stems were washed one by one in water, as shown in Figure 1c, and the fibers were extracted. The general appearance of the extracted fibers is shown in Figure 1d. After the process, the fibers were sent to laboratory centers for relevant tests.

### 2.2. Mechanical, Physical and Chemical Analysis of PoLfs Fiber

The fibers were pulverized by ball-milling prior to chemical analysis. A Fritsch Planetary Micro Mill from PULVERISETTE 7 (Fritsch GmbH, Oberstein, Germany) was used for ball-milling. For five minutes, the fibers were ball-milled at 800 rpm. The powdered stem fibers were dried in an oven at 100 degrees Celsius for four hours to remove excess moisture, which was probably present during both decomposition and washing [37]. Post-dried stem fibers, free of excess moisture, were used for chemical composition analysis. Thus, the cellulose, lignin, wax, and moisture contents in the structure of the fibers were determined. The Mylsamy and Rajendran method was then performed after the drying process [42].

Tensile tests of *Platanus orientalis* L. fruit stem fibers were conducted on 10 mm long fibers at a speed of 0.1 mm/min using an Instron Universal Tester universal testing machine consisting of a 50 N load cell. Under standard atmospheric conditions, every test was carried out in accordance with ASTM D 3822-07 [43] guidelines. Because natural fibers typically have an irregular shape, thirty repetitions of the single fiber test were conducted [44]. The tensile strength and tensile modulus were calculated by Equation (1).

(1) $$\sigma =\frac{F_{b}}{S_{0}}$$

where σ represents the tensile strength and $F_{b}$ and $S_{0}$ denotes max force at fiber break and the cross-sectional area of the fiber, respectively. The tensile modulus and elongation at break were calculated from the test data.

### 2.3. SEM Analysis of PoLfs Fibre

Despite coming from the same plant, natural fibers can differ in their chemical composition, microfibrillar angle, structure, physical attributes, crystalline cellulose diameter, flaws, and isolation method. There may also be notable differences in the mechanical properties and characteristics [31]. Therefore, like many natural fibers, stem fibers have an irregular shape and typically show a heterogeneous cross-section. The accurate measurement of each fiber’s mechanical properties may for that reason become problematic as a result. The diameter measurement was performed by using an SEM-ZEISS/EVO LS10 device from random locations. As per the widely used ASTM D8171-18 [45] standard in the literature, the density measurement of the extracted fiber was performed. Using Archimedes’ principle, the ASTM D8171-18 [45] method measures the buoyancy created by submerged fibers in a fluid. This information allowed for the calculation of the sample’s volume and, consequently, its density [46].

### 2.4. FTIR Analysis of PoLfs Fiber

Fourier Transform Infrared (FTIR) spectra of the stem fibers of the fruit were determined by using a Perkin Elmer Spectrum BX Fourier Transform Infrared Spectrometer (PerkinElmer Life and Analytical Sciences, Bridgeport Avenue Shelton, CT, USA). To record the data in the range of 4000 cm^−1^ to 400 cm^−1^, a signal-to-noise ratio resolution of 2 cm^−1^ and a scan rate of 32 scans per minute were used.

### 2.5. XPS Analysis of PoLfs Fiber

The atomic concentration of the PoLfs fiber was measured using an X-ray photoelectron spectroscopy (Thermo Fisher Scientific Inc., East Grinstead, UK) apparatus that had the characteristics of a monochromatic Al-Ka (1486.7 eV) X-ray source and a 300 µm diameter beam. The range used to gather XPS data was 1361–10 eV, with a precision of 0.1 eV and pass energy of 50 eV. Ionic Ar gas was sputtered before the fiber sample was examined on the surface, and 10 scans were made from a single spot to collect the data.

### 2.6. XRD Analysis of PoLfs Fiber

For determining the crystalline structure of *Platanus orientalis* L. stem fibers, the Rigaku miniflex600 (Rigaku Corp., Tokyo, Japan) device was used. Prior to the measurement procedure, the moisture was extracted for 24 h at 105 °C from the grounded stem fibers. With Cu-Kα radiation (λ-Kα = 1.54 Å) as the X-ray source, the device ran at 40 kV and a 15 mA current. Each sample was scanned at a width of 2θ = 5–55 degrees with a step speed of 0.02° and at a rate of scanning speed of 0.75° per minute (therefore, a total 67 min duration).

A common tool for quantifying the amount of crystalline fraction in cellulosic materials and measuring how much they have changed after undergoing various physicochemical and biological treatments is the crystallinity index (CI) [47]. The crystalline phase content, or CI, is just the percentage that is present. Consequently, determining cellulose structure depends heavily on measuring CI. The data generated by the reflection of the stem fiber’s X-ray diffraction (XRD) pattern were used to calculate the crystallinity index. Segal’s equation in Equation (2) was used for determining the crystalline structure of *Platanus orientalis* L. fruit stem fibers [48]. The 200 peak, located between the scattered angles of 2θ = 22° and 23°, represents the total of the crystalline and amorphous components. The amorphous-only component in this empirical method is represented by the intensity at the minimum, approximately 18°, between the overlapped peaks (1$\bar{1}$0 and 110) and 200 peaks.

(2) $$CI\;\left(\%\right)=\frac{I_{200}-I_{am}}{I_{200}}$$

where $I_{am}$ stands for the 2θ intensity at roughly 18° which is the low value between the overlapped peaks (1$\bar{1}$0 and 110) and (200) peak. $I_{200}$ is associated with the (200) lattice plane between 22° and 23° and shows the peak at maximum intensity [48].

### 2.7. TGA Analysis of PoLfs Fiber

The Seiko SII TG/DTA 7200 (Seiko, Chiba, Japan) instrument was used to measure the TG and DTA of *Platanus orientalis* L. fruit stem fibers in order to evaluate their thermal stability. The chemical stabilization characteristic of the fibers was ascertained by heating 5 mg weights of fiber specimens in a nitrogen environment at a rate of 10 °C per minute from 30 °C to 700 °C.

## 3. Results and Discussion

### 3.1. Mechanical, Physical and Chemical Analysis of PoLfs Fiber

Chemical and physical analysis results of *Platanus orientalis* L. fruits stem fiber and other fibers are presented in Table 1 and mechanical analysis results are presented in Table 2. The mechanical properties of natural fibers and their degradation under environmental conditions are directly related to the chemical composition of the fiber [49,50,51]. The cellulose content of *Platanus orientalis* L. fruit’s stem fiber given in Table 1 is higher than wheat straw, rice straw, rice husk, bamboo and coir fibers and lower than Coccinia indica, Cocos nucifera Peduncle, Cortaderia selloana grass and Sida mysorensis fibers. The mechanical properties of *Platanus orientalis* L. fruit stem fiber with sufficient cellulose content will contribute positively [50,52,53,54,55,56,57,58]. However, the reason why the tensile strength value given in Table 2 is as high as expected is that it is thought to be affected by the cellulose content of 42.03% as well as other chemical contents [49]. Hemicellulose content negatively affects fiber strength as it alters microfibrils. In addition, hemicellulose content can affect thermal resistance, moisture, and biodegradability [51,59]. *Platanus orientalis* L. fruit stem fiber’s 13.5% hemicellulose content is lower than other fibers except for coir, jute and Ageratina Adenophora fibers. Lignin affects the fiber’s moisture absorption and strength properties such as stiffness [49]. *Platanus orientalis* L. fruit’s stem fiber contains lower lignin content (28.35%) than bamboo, coir fibers but higher content than wheat straw, rice straw, rice husk, Ageratina Adenophora, Coccinia indica Cocos nucifera, Peduncle, Cortaderia selloana grass, jute, flax, hemp, kenaf, sisal and Sida mysorensis fibers [50,52,53,54,55,56,57,58].

The chemical contents that make up the structure of natural fibers vary depending on all environmental conditions, such as climate and soil, affecting the plant [50,52,53,54,55,56,57,58]. Therefore, the cellulose ratio, which covers most of the basic chemical content of the fiber, is the most important parameter affecting the mechanical properties of fiber-reinforced composites.

The density of *Platanus orientalis* L. fruit stem fiber was determined as 1.36 (g/cm^3^). It is seen that this is similar to the density values of other natural fibers given in Table 1. In fiber-reinforced composites, low-density fibers are more advantageous than higher-specific-gravity fibers because they contribute to the production of lightweight materials [51].

*Platanus orientalis* L. fruit’s stem fiber has moisture content of 10.86%, which is higher than the bamboo, coir, Ageratina Adenophora, Coccinia indica and Cortaderia selloana grass fiber values given in Table 1. Low moisture content is desired in fiber-reinforced composites. Plane-tree-fiber-reinforced composites should pay attention to the removal of moisture content for better fiber/matrix surface compatibility [52,53,54,55,56,57,58,59,60,61,62,63,64,65].

**Table 2:** Mechanical properties of *Platanus orientalis* L. fruit stem fiber and other natural fibers [37,44,58,60,66,67].

Fiber	Tensile Strength (MPa)	Tensile Modulus (GPa)	Elongation (%)
*Platanus orientalis* L. fruit’s stem	157.76 ± 23	1.39 ± 0.42	22.01 ± 3.7
Oil Palm	80–248	0.5–3.2	17–25
Feather	100–203	3–10	6.9
Coir	135–240	4–6	15–40
Bagasse	222–290	17–27.1	1.1
Banana	1.7–7.9	-	1.5–9.0
Date palm	90–176	3–7.7	3.8–4.8
Chrysanthemum morifolium	65.12	1.55 ± 0.7	4.51 ± 0.95
Napier grass	88.40	13.1	0.99
Veldt-grape stem fiber	61.42	1.1	5.6
Hierochloe Odarata	105.73	2.56	2.37
Glycyrrhiza glabra	132.40	4.47	4.48
Jute	393–773		1.5–1.8
Flax	345–2000		1–4
Hemp	368–800		1.6
Kenaf	240–930		1.6
Sisal	350–700		2–7
Ramie	400–1000		1.2–3.8

The fineness value of the obtained fibers was obtained as 181.01 ± 9.81 μm. When this value is compared with the average of the fineness values of traditional natural fibers quoted in the literature, it is seen that this value is smaller than kenaf, ramie, jute and sisal fibers, but larger than hemp and flax fibers.

Scanning electron microscope images were used to determine the diameter of *Platanus orientalis* L. fruit stem fiber. The average of 15 measurements was taken from the surface of different fibers and the diameter was determined as 181.01 ± 9.81 μm. The average fiber length of 15 measurements was determined to be 12.43 ± 2.6 cm. The physical properties of natural fibers generally vary depending on their age and type [35,58,68]. Fiber diameter is used to calculate the tensile strength value by determining the fracture surface area. In the literature, it has been determined that the cellulose ratios of the fibers obtained from different parts of the plant such as roots, stems and leaves vary, and this obviously affects the mechanical properties [69]. The mechanical properties of the fiber can be improved by removing non-cellulose contents with chemical surface treatment applications [25,38,64,70].

The tensile strength value of *Platanus orientalis* L. fruit’s stem fiber was found to be 157.76 ± 23 MPa. The tensile modulus value was 1.39 ± 0.42 GPa, and the elongation value was 22.01 ± 3.7%. It was found to have a higher tensile strength value than Chrysanthemum morifolium Napier grass, Veldt-grape stem fiber, Hierochloe Odarata and Glycyrrhiza glabra fibers, shown in Table 2, and a lower value than Bagasse fiber. In addition, the tensile modulus value was low due to the high lignin ratio, which is one of the characteristic features of stem fibers, but the elongation value was higher than the other fibers except for Oil Palm fiber [37,44,58,66,67].

It was concluded that *Platanus orientalis* L. fruit’s stem fiber can improve the mechanical properties of a material by using it as a reinforcing fiber in composite and biocomposite applications.

### 3.2. SEM Analysis of PoLfs Fibre

**Figure 2:**
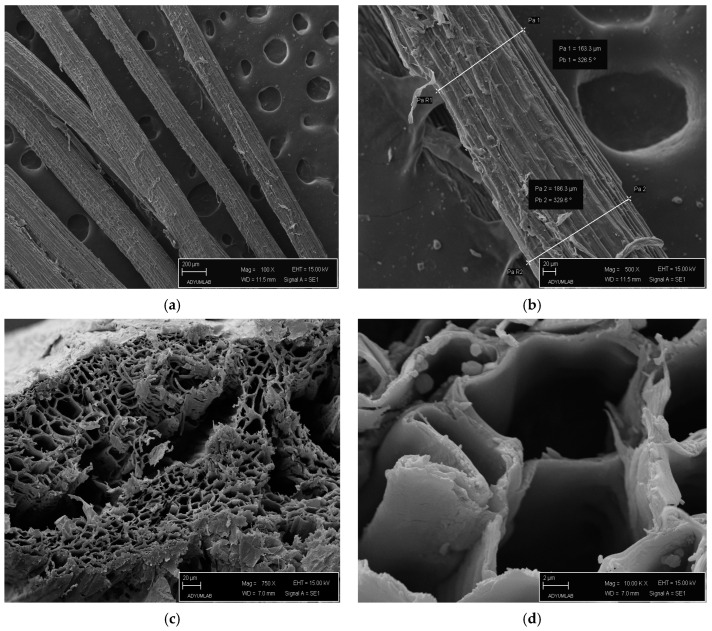
(**a**,**b**) Longitudinal and (**c**,**d**) transverse section images of *Platanus orientalis* L. fruit’s stem fiber.

SEM images of *Platanus orientalis* L. fruit’s stem fiber at 100×, 500×, 750× and 1000× biomass are given in Figure 2. The surface of PoLfs fiber showed roughness and ripples. Figure 2a shows a longitudinal section obtained at 100× magnification, which shows a complex arrangement of microfibrils, lignin and non-cellulosic impurities [49]. The image obtained at 500× magnification Figure 2b shows cellulose microfibrils and lignin [71,72].

In addition, agglomerates of amorphous substances or impurities, including hemicellulose and lignin, were observed on the surface of the fiber, which did not resemble a specific geometric shape [73]. In addition, the wavy and rough surface structure of the surface structure can be an advantage in composite contexts. It can contribute positively to interfacial compatibility by mechanically combining the fibers with the matrix [32,72].

### 3.3. FTIR Analysis of PoLfs Fiber

**Figure 3:**
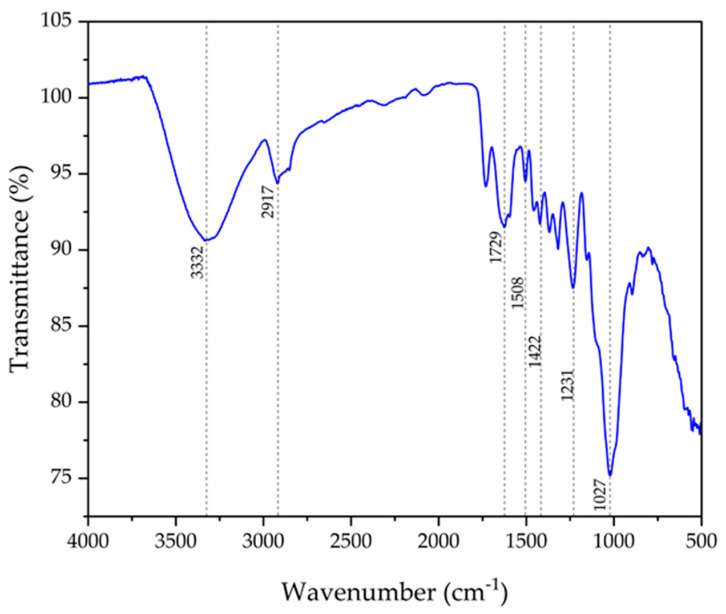
FTIR spectrum of PoLfs fibers.

The FTIR curve of *Platanus orientalis* L. fruit’s stem fiber is given in Figure 3. It can be seen that 3332 cm^−1^ is the broad peak showing hydroxyl groups, indicating the presence of cellulose, lignin and water [71]. The peaks at 2917 and 1729 cm^−1^ correspond to alkanes (C-H) and a carboxyl group (C=O) attributed to cellulose and hemicellulose [70]. The peak at 1625 cm^−1^ represents the (C=C) stretching of lignin [74]. The peaks between 1408 and 1163 cm^−1^ are related to the C=O stretching of hemicellulose, lignin and the carboxyl group. The presence of polysaccharides in the structure of cellulose corresponds to the stretching vibration at 1027 cm^−1^ [49].

### 3.4. XPS Analysis of PoLfs Fiber

**Figure 4:**
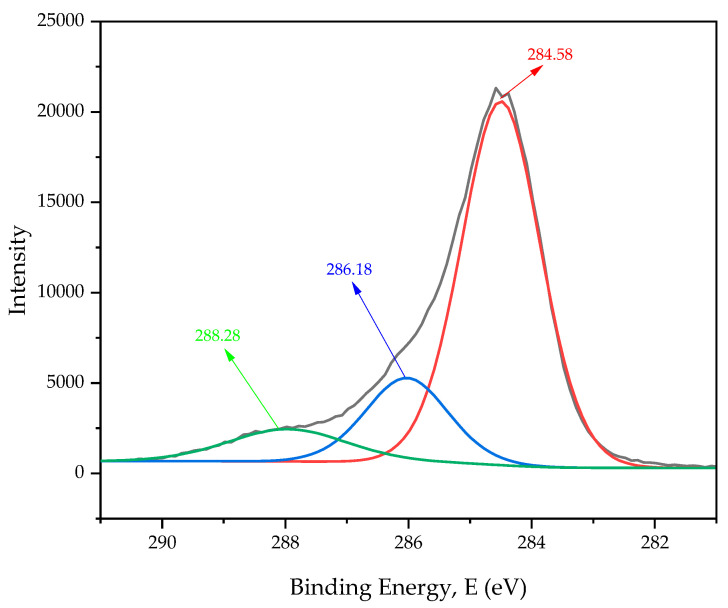
C1s XPS spectrum peaks of PoLfs.

The carbon and oxygen ratios of *Platanus orientalis* L. fruit stem fiber are given in Table 3. The C1 spectrum determined by XPS analysis is given in Figure 4. The carbon and oxygen ratios of *Platanus orientalis* L. fruit’s stem fiber were determined as 71.94% and 14.7%, and all other ratios are given in Table 3.

Carbon/oxygen (C/O) (4.89%) and oxygen/carbon (O/C) (0.20%) ratios were calculated to determine the surface morphology of the fiber. In cellulosic-based fibers, the C/O ratio is related to the hydrophobic property of the surface, and this property must be known for its suitability for use in fiber-reinforced composites [44]. *Platanus orientalis* L. fruit’s stem fiber (4.89%) was found to have a higher carbon/oxygen ratio and a more hydrophobic surface character than jute (2.09%) and kenaf (2.38%) fibers, known to be used in composites.

The peak at 284.58 in the C1s spectrum shown in Figure 4 corresponds to C-C/C-C-H and O=C groups representing the presence of cellulose or ether [75]. The peak at 286.18 represents carbonyl groups (C=O/O-C-O), and the peak at 288.28 represents O-C=O, indicating lignin and carboxylic acid [76].

### 3.5. XRD Analysis of PoLfs Fiber

**Figure 5:**
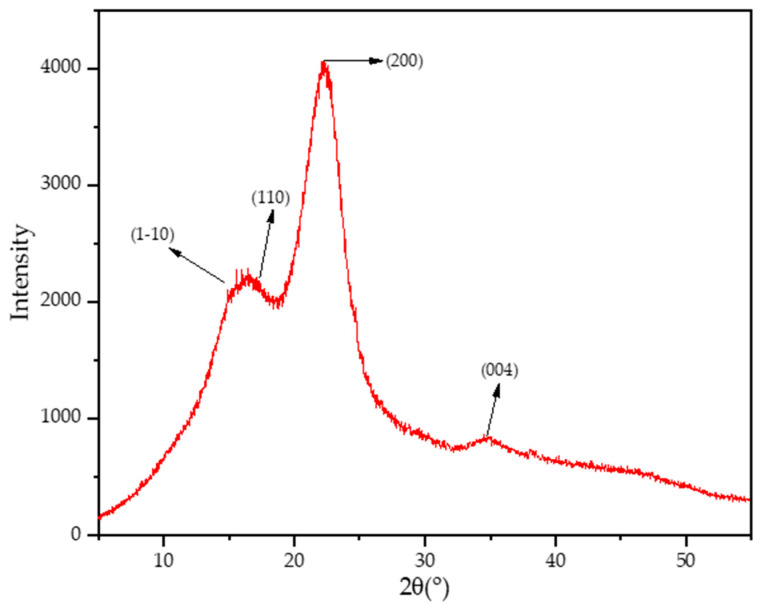
XRD pattern of PoLfs.

The XRD curve of *Platanus orientalis* L. fruit’s stem fiber is shown in Figure 5. The first peak at 15.74° corresponds to cellulose I formed by the overlap of the (1$\bar{1}$0) and (110) lattice planes [44,75,77]. The large peak at 20.21 indicates the (200) lattice plane, which is the characteristic peak of cellulose I [44,75,78]. The low peak in the region between 16 and 22 was observed at 18 and is associated with the amorphous structure of the fiber [79].

This result, obtained by XRD analysis, coincides with the results of chemical and FTIR analysis, indicating the presence of cellulose and amorphous structures. The crystallinity index value of *Platanus orientalis* fruit’s stem fiber was calculated as 52.16% with the Segal Equation [49]. The crystallinity index value of *Platanus orientalis* fruit’s stem fiber is higher than the value of some of the fibers in the literature such as Pandanus amaryllifolius (37.09%), Cymbopogan citratus (35.20%), Tridax procum-bens (34.46%), Juncus effuses L. (33.40%) and Ficus Religiosa (42.92%) [49,80,81,82,83,84,85]. Fibers with high crystallinity index values can increase the mechanical properties of composites [44]. *Platanus orientalis* L. fruit’s stem fiber with a 51% crystallinity index value was found to be a suitable alternative to be utilized in the fiber-reinforced composite industry.

### 3.6. TGA Analysis of PoLfs Fiber

**Figure 6:**
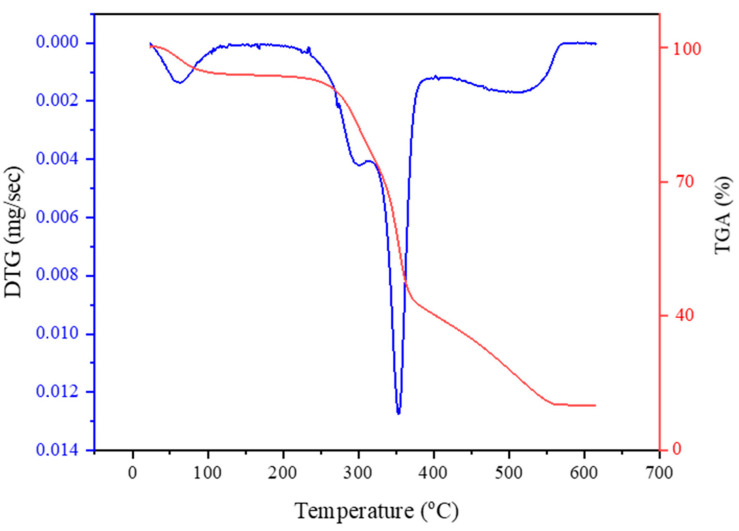
TGA-DTG curve of PoLfs fiber.

Figure 6 shows the thermogravimetric analysis curve of *Platanus orientalis* fruit’s stem fiber. With TGA analysis, the reaction of the fiber against heat is determined. The first degradation occurred between 0 and 100 °C with the evaporation of water in cellulosic fibers [67]. The second decomposition was observed at 296.84 °C, and this is the temperature at which the fiber can burn without decomposing in the face of heat. After this temperature, cellulose and hemicellulose start to decompose [78]. The third degradation was detected at 353.56 °C. At this temperature, cellulose decomposition is complete, and this indicates the maximum temperature of the fiber [83]. The degradation of lignin and other substances takes place at 400–550 °C. At the last temperature value at 620 °C, it was found that about 12.86% of the lignin left a residue in the form of charcoal. It is important to determine the thermal resistance of the fiber in natural fiber composites [83]. *Platanus orientalis* L. fruit’s stem fiber was found to be suitable for composite production at temperatures below 296.84 °C.

## 4. Conclusions

The suitability of *Platanus orientalis* fruit’s stem fiber for composite applications has been investigated by characterization with chemical analysis, FTIR, XRD, XPS, SEM, TGA and fiber density analysis. *Platanus orientalis* L. fruit’s stem fiber was determined to be suitable for fiber-reinforced composite production with a cellulose ratio of 42%. A 71.94% C/O ratio, which is higher than some other natural fibers, could cause an increase in the hydrophobic character of the surface. A 52.16% crystallinity index value was calculated by using Segal’s method. Mechanical properties, i.e., the tensile strength, tensile modulus and elongation value, were calculated as 157.76 ± 23 MPa, 1.39 ± 0.42 GPa and 22.01 ± 3.7%, respectively. It was determined that PoLfs fiber is suitable for utilization in fiber reinforcement in thermoplastic-based composites at temperatures below 299 °C. According to the results obtained by the mechanical, chemical and physical analysis of *Platanus orientalis* L. fruit’s stem fiber, it could be recommended as a suitable alternative as a reinforcing fiber in thermoplastic and thermoset composites. For future studies, the chemical treatment of extracted light fiber to improve properties and measurements to evaluate compatibility with the polymer matrix will be addressed.

## Figures and Tables

**Table 1:** Comparison of chemical ratios and density of *Platanus orientalis* L. fruit’s stem fiber and other natural fibers [50,52,53,54,55,56,57,58,60,61].

Natural Fibers	Density (g/cm^3^)	Fineness (Micron)	Moisture Content (%)	Cellulose (%)	Hemicellulose (%)	Lignin (%)
PoLfs	1.36	181.01 ± 9.81	10.86	42.03	13.5	28.35
Bamboo	0.910		9.16	26–43	30	1–31
Coir	1.15		8	32–43	0.15–0.25	40–45
Wheat straw	-		-	38–45	15–31	12–20
Rice straw	-		-	41–57	33	8–19
Rice husk	-		-	35–45	19–25	20
Ageratina Adenophora	1.32		7.4	65.7	11.2	12.5
Coccinia indica	1.37		7.27	64.56	14.09	12.55
Cocos nucifera Peduncle	1.3–1.4		11.1	50.1	24.9	11.9
Cortaderia selloana grass	1.26		7.6	53.7	14.43	10.32
Sida mysorensis	1.29		10.48	53.36	15.23	9.46
Jute	1.3	25–200		64.4	12	11.8
Flax	1.5	40–600		64.1	16	2.0
Hemp	1.47	25–500		68	15	10
Kenaf	1.45	12–36		31–72	20.3–21.5	8–19
Sisal	1.5	25–200		60–78	10–14.2	8–14
Ramie	1.5	25–50		68.6–85	13–16.7	0.5–0.7

**Table 3:** Atomic constituents forming the surface of *Platanus orientalis* L. fruit’s stem fiber.

	Cls (%)	O1s (%)	N1s (%)	C/O (%)	O/C (%)
*Platanus orientalis* L. fruit’s stem	71.94	14.7	1.8	4.89	0.20

## Data Availability

The related data as discussed in this article can be requested from the corresponding author.
